# Significant Cellular Viability Dependence on Time Exposition at ELF-EMF and RF-EMF In Vitro Studies

**DOI:** 10.3390/ijerph16122085

**Published:** 2019-06-13

**Authors:** Olga García-Minguillán López, Ana Jiménez Valbuena, Ceferino Maestú Unturbe

**Affiliations:** 1 CTB (CTB-UPM) Centro de Tecnología Biomédica, Universidad Politécnica de Madrid, 28223 Pozuelo de Alarcón, Spain; olga.garcia-minguillan@ctb.upm.es; 2 Escuela Técnica Superior de Ingenieros de Telecomunicación, Universidad Politécnica de Madrid, 28040 Madrid, Spain; ana.jimenez.valbuena@alumnos.upm.es; 3 CIBER-BBN Centro de Investigación Biomédica en Red, 28029, Madrid, Spain

**Keywords:** ELF-EMF, RF-EMF, glioblastoma, fibroblast, Helmholtz coil, Gtem

## Abstract

The human concern about the effect of electromagnetic fields (EMFs) has changed over time from the effects produced by EMFs of extremely low frequencies (ELFs) to the effects produced by exposure to a radio frequency (RF), with concerns shifting toward EMFs due to the development of new technologies and forms of communication. Previous studies have analysed the effects produced at different frequencies without considering in detail the effect of the time of exposure. Therefore, in the present study, we analysed in vitro the effect produced by a 100 µT EMF at different ELFs and exposure times in glioblastomas, as well as the effect produced in a fibroblast by an RF-EMF of 2.54 GHz. Our results indicate a significant time dependence in cell viability of fibroblasts exposed to an RF-EMF of 2.54 GHz and a non-time-dependent effect in cell viability of glioblastomas exposed to an ELF-EMF, highlighting the possible relation between frequency and time of exposure.

## 1. Introduction

Since the industrial revolution, human concerns about the effect of electromagnetic fields (EMFs) have changed. Initially, the main concern was the effect produced by EMFs of extremely low frequencies (ELFs) in the range of 1–300 Hz [1]. Currently, due to the development of new technologies and novel forms of communication, such as Wireless Fidelity (Wi-Fi), the concerns about the effect produced by exposure to EMFs also include extremely high frequencies (EHFs).

The concern about exposure to EMFs is the possible harmful effects on humans even when the field is under the security limits established by the International Commission on Non-Ionizing Radiation Protection (ICNIRP). Regarding the effect of ELF-EMFs, one of the largest studies that has been done is the multicounty project called Risk Evaluation of Potential Environmental Hazards From Low Energy Electromagnetic Field Exposure Using Sensitive in vitro Methods (REFLEX), where the genotoxicity, cell proliferation, and apoptosis of different cell lines, including fibroblasts and neuroblastomas, were analyzed [2]. The REFLEX project revealed differences in the effect produced by ELF-EMFs depending on such parameters as the time of exposure, the frequency, and the cell line.

Following the results obtained in the REFLEX project, several in vitro studies on ELF-EMFs have been done in different cancerous and noncancerous cells, supporting the fact that ELF-EMFs produce changes in cell viability [3], with several of those studies focused on the effects produced on neuroblastomas and glioblastomas [4,5].

On the other hand, regarding the effect produced by EHF-EMFs, the REFLEX Project found genotoxic effects on, and chromosomal aberration in, fibroblasts exposed to radio frequency (RF) EMFs [2]. Additionally, other in vitro studies have found, under no thermal effects, changes in cell apoptosis in primary cultures of neurons and astrocytes [6], increases in cell proliferation in human astrocytoma cells [7], inhibition of the formation of p53 binding protein 1 (53BP1) in a primary culture of human fibroblasts [8] and a possible activation of fibroblast proliferation [9].

For these reasons, the exposure limits for EMFs have remained unchanged since 1999. Namely, the recommended limits of exposure are a magnetic flux density equal to 2400 divided by a frequency for ELF-EMFs between 8 and 25 Hz, a magnetic flux density equal to 20 divided by a frequency for ELF-EMFs between 25 and 82 Hz and a power density of 50 W/m2 for EHF-EMFs with frequencies between 200 and 300 GHz [10].

Due to this situation, in the present study, the effect produced on glioblastomas by EMFs at the recommended exposure limits was analysed in detail at different ELFs (20 Hz, 30 Hz and 50 Hz), since most of the research done previously has considered EFL-EMFs whose frequencies exceed the recommended limit [11]. Furthermore, the present research analyses the non-thermal effect produced by an EHF-EMF of 2.54 GHz on fibroblast viability by using a complex system called a gigahertz transverse electromagnetic (GTEM) cell, which allows us to perform emission tests in a completely controlled environment, complementing previous research conducted on other types of exposure systems.

## 2. Materials and Methods

### 2.1. Cell Lines and Cell Culture

The mouse glioma cell line CT2A and the mouse embryonic fibroblast cell line NIH/3T3 were maintained at 37 °C and in 5% CO_2_. The CT2A cell line was grown in RPMI medium (Solmeglas w/L glutamine, w/ 25mM Hepes) supplemented with 10% Fetal Bovine Serum, 1% L-glutamine and 1% streptomycin and the NIH/3T3 cell line was grown in DMEN medium (Biowest w/o L-glutamine, w/ 25 mM Hepes, w/o sodium pyruvate) supplemented with 10% Fetal Bovine Serum, 1% L-glutamine and 1% streptomycin. For all of the experiments at extreme low frequency, CT2A cells were seeded in p35 cell culture dishes in a final concentration of 175,000 cells/ml 24 h before exposure. For all of the experiments at extreme high frequencies, NIH/3T3 cells were seeded in p35 cell culture dishes in a final concentration of 60,000 cells/ml 24 h before exposure.

### 2.2. Exposure to Electromagnetic Fields with Extreme Low Frequencies Between 20 Hz and 50 Hz

The EMF was generated by using a pair of Helmholtz coils of 19 turns, each one separated by a distance of 12 cm, equal to their radius. The configuration of the Helmholtz coils allows for them to generate a homogeneous EMF in the center of their axis. Therefore, the culture dishes were placed 6 cm away from each coil.

**Figure 1:**
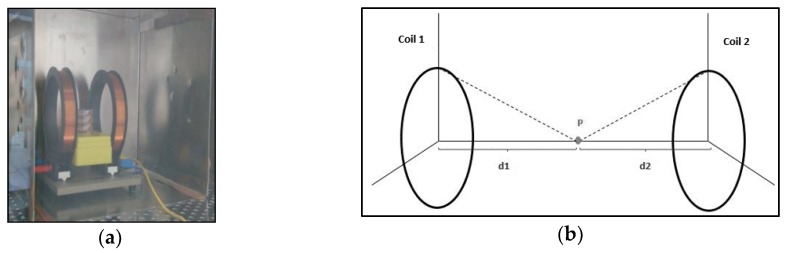
The extremely low frequencies-electromagnetic field (ELF-EMF) system: (**a**) a pair of Helmholtz coils inside a mu-metal box; (**b**) the scheme of a pair of Helmholtz coils, where P is the point placed in the center of the axis, d1 is the distance from Coil 1 to the point P, and d2 is the distance of the Coil 2 to the point P.

Cells were exposed at 20 Hz, 30 Hz and 50 Hz for 24 h, 48 h and 72 h to an EMF of 100 µT, which is the maximum recommended EMF value established by Council Recommendation 1999/519/EC for a frequency of 50 Hz. To avoid external EMFs, the coils with the cells were placed in an incubator inside a mu-metal box (Figure 1).

The exposure frequencies were controlled by a laboratory Frederiksen function generator, and the value that the EMF generated was set up by an FAC 662B Promax power supply. The homogeneity of the EMF was generated by the pair of coils and measured by a LakeShore-Cryotronics 450 gaussemeter, both in the center of the culture dishes and in their extremes. A mean value of 99.99 µT ± 6.81 standard error was obtained.

Control cells were maintained under the same conditions and for the same times with no EMF exposure (Helmholtz coils off).

### 2.3. Exposure to Electromagnetic Fields with Extreme High Frequencies

The EMF was generated using a gigahertz transverse electromagnetic (GTEM) cell model 250 TESEQ, which is a test chamber that is designed to execute a compatibility electromagnetic test without external interference.

Cells were exposed inside the GTEM cells for 15 min, 3 h, 6 h, 9 h, 15 h, 18 h and 21 h to 56.2 µW/cm^2^ under non-thermal effects at a fixed frequency of 2.54 GHz. The field created inside the GTEM was controlled by a Giga-tronics 2520 external function generator, a Microwave Synthesizer and a Giga-tronics 1000 A Microwave Power Amplifier (Giga-tronics, Dublin, CA, USA).

**Figure 2:**
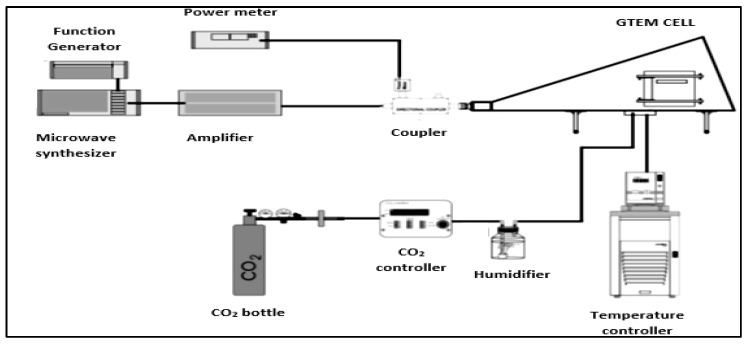
The general scheme of the complete gigahertz transverse electromagnetic (GTEM) assembly.

**Figure 3:**
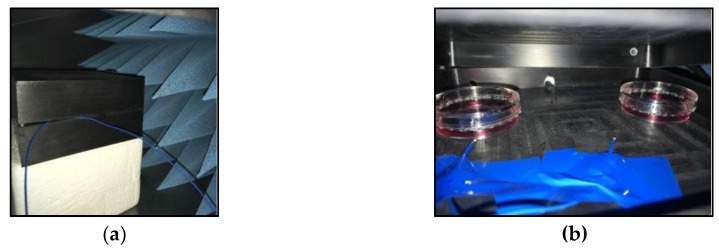
(**a**) The EUT chamber where the cells were placed; (**b**) NIH/3T3 mouse fibroblast cell line cells inside the EUT chamber.

The physiological conditions required by the cells, 37 °C and 5% of CO_2_, were set up by a Julabo F25-ME external criothermostat (Julabo, Seelbach, Germany) that was connected to a water bath and an Olympus CTI_Controller 3700 external CO2 regulator, which was connected to a CO_2_ bottle (Figure 2 and Figure 3).

The intensity of the electromagnetic field, the input power and the power density were measured by a Rohde Schwarz spectrum analyser (Rohde Schwarz, Munich, Germany) connected to an isotropic antenna.

Control cells were maintained under the same conditions and for the same times with no EMF exposure (Gtem off).

### 2.4. Cell Viability

2,3-Bis-(2-Methoxy-4-Nitro-5-Sulfophenyl)-2H-Tetrazolium-5-Carboxanilide (XTT) assay.

The viability of CT2A cells after exposure to an ELF-EMF was measured by the XTT coulometric assay. The XTT assay (Cell Proliferation Kit XTT PanReac AppliChem) was performed by adapting the instructions of the manufacturer. To each well of a 24-well culture plate containing 234 µL of medium, 114 µL of XTT reagent was added as well as 2 µL of activation solution. The cells were incubated with XTT for 1 h at 37 °C and in 5% CO_2_, and the absorbance was measured with a spectrophotometer at a wavelength of 450 nm.

### 2.5. Trypan Blue Assay

The viability of the NIH/3T3 cells after the exposure to an EHF-EMF was measured by the trypan blue dye. After exposure, the cells were briefly detached from the culture dishes by incubating them for 5 min at 37 °C and in 5% CO_2_ with trypsin, and centrifugation for 5 min at 1200 rpm and 21 °C. The supernatant was then discarded and the pellet was resuspended in 1 mL of medium. Subsequently, the concentration of cells was measured by mixing 50 µL of cells with 50 µL of trypan blue and counting them in a Neubauer chamber.

The percentage of alive cells was computed as follows (Equation 1): The percentage of alive cells by a trypan blue dye exclusion test.

(1) $$\%\;alive\;cell=\left(\frac{number\;of\;alive\;cells}{number\;of\;alive\;cells\;plus\;number\;of\;dead\;cells}\right)\times 100$$

### 2.6. Statistical Analysis

A Student’s *t*-test at a confidence level of 95% was performed for all the ELF-EMF experiments to compare the viability of the CT2A control group (Helmholtz coils off) and the viability of CT2A cells exposed to different time periods and frequencies.

A Student’s *t*-test at the confidence level of 95% was also performed for all the RF-EMF experiments to compare the viability of the NIH/3T3 control group (GTEM off) and the viability of NIH/3T3 cells exposed to different periods of time.

## 3. Results

### 3.1. Time and Frequency Results

#### 3.1.1. Time and Frequency Effects of a 100 µT ELF-EMF on Cell Viability

**Figure 4:**
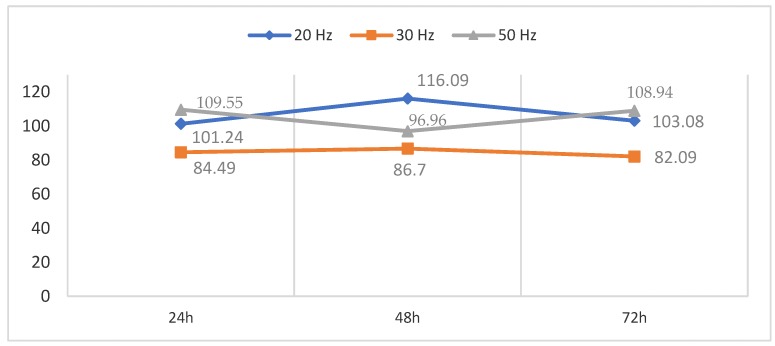
Percentage of viable CT2A cells after exposure to a 100 µT EMF at 20 Hz, 30 Hz and 50 Hz for 24 h, 48 h and 72 h.

The different effects produced on CT2A cell viability after exposure to a pulsed 100 µT EMF at 20 Hz, 30 Hz and 50 Hz for 24 h, 48 h and 72 h were measured by the XTT assay (Figure 4). The results show a decrease in viability of the CT2A cells exposed for 24 h, 48 h and 72 h to a 100 µT EMF at 30 Hz in the control group (Helmholtz coils off). On the other hand, the CT2A cells exposed for 24 h, 48 h and 72 h to a 100 µT EMF at 20 Hz showed an increase between 1.06% and 16.12% in contrast to the control group. Finally, the CT2A cells exposed to a 100 µT EMF at 50 Hz showed an increase in cell viability of 10.77% and 8.94% for the exposure time of 24 h and 72 h, respectively, and a decrease of 4.95% for the exposure time of 48 h.

The *p*-values of the results are as follows (Table 1, Table 2 and Table 3):

#### 3.1.2. Time-Dependent Effect of an EHF-EMF on Cell Viability

**Figure 5:**
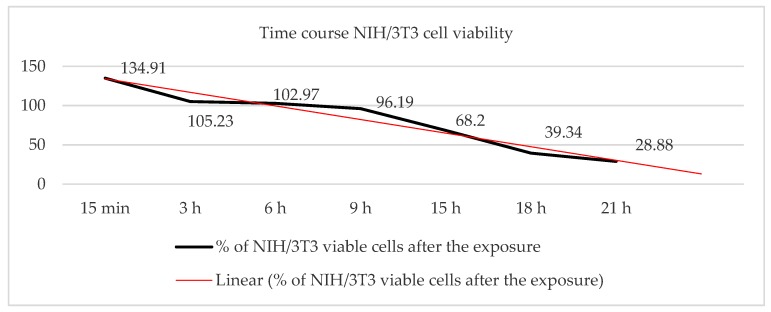
Percentage of viable NIH/3T3 cells after exposure to a radio frequency (RF)-EMF of 56.20 µW/cm^2^ at 2.54 GHz for 15 min, 3 h, 6 h, 9 h, 18 h and 21 h.

The effect produced on NIH/3T3 cell viability after exposure to a 56.2 µW/cm^2^ EMF at 2.54 GHz for 15 min, 3 h, 6 h, 9 h, 15 h, 18 h and 21 h was measured by the trypan blue exclusion dye test (Figure 5). The results show an increase in viability between 34% and 2.97% among the NIH/3T3 cells that were exposed to the EMF for a period of 15 min, 3 h and 6 h compared to the control group (GTEM off).

However, after the first six hours, the results showed a 4% viability decrease in the NIH/3T3 cells exposed to the EMF for 9 h, a decrease of 32% in the cells exposed to the EMF for 15 h and a decrease of 61% in the viability of the cells exposed to the EMF for 21 h.

## 4. Discussion

In the present study, we analysed the effect produced either by an EMF of 100 µT at different ELFs (20 Hz, 30 Hz and 50 Hz) or an RF-EMF of 56.2 µW/cm^2^ at 2.54 GHz on cell viability in two different cell lines: CT2A mouse glioma cells and NIH/3T3 mouse embryonic fibroblasts.

In accordance with our data, an EMF of 100 µT at a frequency of 50 Hz for 24 h and 72 h of exposure produces an increase in CT2A viability (*p* < 0.05), which does not vary with time of exposure (Figure 4). These results seem to be consistent with the results of a previous study, where an increase in proliferation of SH-SY5Y human neuroblastomas was found after exposure to an EMF of 1 mT at 50 Hz [12], but inconsistent with the results of another study [13] where a decrease in NIH/3T3 cell viability was found after exposure to an EMF of 1 mT at 60 Hz.

Nevertheless, we also found that, after 24 h, 48 h and 72 h of exposure to a 20 Hz EMF of 100 µT, an increase in the cell viability of CT2A cells was produced. However, the difference in cell viability from the control group (Helmholtz coils off) at 24 h and 72 h can be neglected (*p* > 0.05). Paradoxically, a time of exposure of 48 h for an EMF of 100 µT at a frequency of 50 Hz according to our results did not affect the relative cell number (*p* > 0.05), which suggests that this result could align with the hypothesis that the effects of EMF are directly related to such parameters as the time, frequency and amplitude of the EMF [5].

Our main hypothesis is that cell variability is correlated with frequency and time of exposure. We found that an ELF-EMF of 100 µT at 30 Hz produces a decrease in CT2A cell viability (Figure 4), although we also found that, at 50 Hz, cell viability did not vary with time of exposure.

Considering all of the above, our results show that an ELF-EMF at the recommended exposure level affects cell viability.

Additionally, our results show a change in the cell viability in NIH/3T3 fibroblasts exposed to an RF-EMF of 56.20 µW/cm2 at 2.54 GHz for 15 min, 3 h, 6 h, 9 h, 18 h and 21 h (Figure 5). In that case, contrary to the results for the ELF-EMF, our results indicate a decrease in viability that increases significantly with the time of exposure, as was described previously in human-adipose-derived stroma cells (ADSC) exposed to an EMF of 354.6 µW/cm^2^ at 900 MHz [14]. 

Previous studies have also found an induction of oxidative DNA damage in mouse-spermatocyte-derived cells exposed to an EMF of 1800 MHz [15], although others have concluded that there is no effect on the progressive motility of human spermatozoa exposed to a pulsed EMF of 900 Hz [16].

These discrepancies could suggest what the REFLEX Project showed: the effect produced by an EMF depends on the cell line [2]. In addition, a recent study done with RF-EMF exposure by the National Toxicology Programm (NTP) showed also sex and species dependence in tumour development, between female and male rat and mice exposed [17].

Therefore, although the pattern of the effect remains unknown, there is clear evidence that RF-EMF produces changes in vitro and in vivo, and, in our case, there is no doubt that a significant cellular decrease occurs after 6 hours of exposure in NIH/3T3 cells (Figure 5), although it is not clear whether the decrease in cell viability is related to an increase in mortality or to the antiproliferative properties of the EHF-EMF.

In spite of previous in vitro studies, our finding with NIH/3T3 cells cannot be completely compared with previous RF-EMF exposure results, given that few studies have been performed using a certified testing chamber, such as the GTEM cell [6], and no recent studies have been done in vitro with fibroblasts at a frequency of 2.54 GHz.

For these reasons, it is not enough to investigate the cellular response of other cancerous and non-cancerous cell lines at different power densities and frequency, but also according to our results is fundamental the exposition time.

## 5. Conclusions

In most of the in vitro studies that have been done with EMFs, the time of exposure has not been considered in detail, even though cellular responses, such as cell proliferation, viability and death, clearly vary between chronic and acute exposure.

Our results demonstrate a time dependence of NIH/3T3 cell viability when exposed to an RF-EMF of 2.54 GHz, which decreases with time to obtain a 71.12% decline in viability after 21 h of exposure. 

On the other hand, our results for an ELF-EMF of 100 µT have demonstrated non-time-dependent effects. Frequency dependence effects were observed, obtaining a primary increase in cell viability at 50 Hz and a reduction in cell viability at 30 Hz, showing that even the recommended exposure level affects cell viability.

Therefore, the relationship between the frequency and time of exposure could determine the cellular response, demonstrating a need for further research.

## Figures and Tables

**Table 1:** The *p*-value of the percentage of viable mouse glioma cell line CT2A cells after exposure to a 100 µT electromagnetic field (EMF) at 20 Hz.

20 Hz	*p* Value
24 h	0.435
48 h	0.005
72 h	0.207

**Table 2:** The *p*-value of the percentage of viable CT2A cells after exposure to a 100 µT EMF at 30 Hz.

30 Hz	*p* Value
24 h	0.007
48 h	0.002
72 h	0.001

**Table 3:** The *p*-value of the percentage of viable CT2A cells after the exposure to a 100 µT EMF at 50 Hz.

50 Hz	*p* Value
24 h	0.009
48 h	0.299
72 h	0.002
